# Detecting Patient Safety Errors by Characterizing Incidents Reported by Medical Imaging Staff

**DOI:** 10.3389/fpubh.2022.846604

**Published:** 2022-03-18

**Authors:** Tarja Tarkiainen, Sami Sneck, Marianne Haapea, Miia Turpeinen, Jaakko Niinimäki

**Affiliations:** ^1^Research Unit of Medical Imaging, Physics and Technology, Oulu University Hospital, University of Oulu, Oulu, Finland; ^2^Administrative Centre, Oulu University Hospital, Oulu, Finland; ^3^Medical Research Centre, Oulu University Hospital, University of Oulu, Oulu, Finland; ^4^Administrative Centre, Research Unit of Biomedicine, Oulu University Hospital, University of Oulu, Oulu, Finland

**Keywords:** incident reporting, medical imaging, errors, patient safety, risks

## Abstract

The objectives of the study were to characterize events related to patient safety reported by medical imaging personnel in Finland in 2007–2017, the number and quality of reported injuries, the risk assessment, and the planned improvement of operations. The information was collected from a healthcare patient safety incident register system. The data contained information on the nature of the patient safety errors, harms and near-misses in medical imaging, the factors that lead to the events, the consequences for the patient, the level of risks, and future measures. The number of patient safety incident reports included in the study was 7,287. Of the incident reports, 75% concerned injuries to patients and 25% were near-misses. The most common consequence of adverse events and near-misses were minor harm (37.2%) related to contrast agent, or no harm (27.9%) related to equipment malfunction. Supervisors estimated the risks as low (47.7%) e.g., data management, insignificant (35%) e.g., verbal communication or moderate (15.7%) e.g., the use of contrast agent. The most common suggestion for learning from the incident was discussing it with the staff (58.1%), improving operations (5.7%) and submitting it to a higher authority (5.4%). Improving patient safety requires timely, accurate and clear reporting of various patient safety incidents. Based on incident reports, supervisors can provide feedback to staff, develop plans to prevent accidents, and monitor the impact of measures taken. Information on the development of occupational safety should be disseminated to all healthcare professionals so that the same mistakes are not repeated.

## Introduction

The World Health Organization (WHO) has constructed global priorities for research to collect information on patient safety incidents. The focus is on the underlying processes and organizational factors that lead to unsafe care and adverse events (1, 2). The goal is to help organizations prevent and reduce the occurrence of serious incidents (3). However, it is well-known that although information on incidents is collected, some are always underreported, under-analyzed, and under-utilized (4–7).

Countries use different methods to investigate and analyze healthcare incidents. For example, the USA uses the root case analysis method of the Joint Commission (8). At the beginning of 2000, the development of patient safety incident reporting systems was initiated in 13 European countries: Austria, Belgium, the Czech Republic, Denmark, Ireland, France, the Netherlands, Norway, Scotland, Spain, Sweden, Switzerland, and the United Kingdom. These countries collect information on patient safety incidents on three different levels: only sentinel events (based on law), specific clinical domains, or healthcare system-wide (including near-misses) (9).

Many countries have their own national and local error reporting systems aiming to identify gaps in the system. If events are only known to individuals, countermeasures cannot be developed. The data, especially on medication-related incidents, are collected from the Medication Error Reporting System (MERS), which is used in the United Kingdom and the United States (4, 9, 10). However, the role of medical imaging in these incidents is not so well-known (2). Australia and New Zealand use a database called the Radiology Events Register (The RaER). Reporting incidents on the RaER is web-based, voluntary, confidential, and anonymous. The system collects information *via* narrative texts about events, their outcomes and contributing factors, and how to prevent or reduce them. The RaER does not replace state and hospital-based incident reporting, but it has been declared a quality assurance resource (2, 11).

In 2005 in Finland, the Ministry of Social Affairs and Health launched national-level coordination and strategic guidance for patient safety measures. A steering group focused on three areas of patient safety: education and culture, tools, and reporting (9). The Finnish healthcare system is based on municipal primary care (about 190 units), specialized care (20 hospital districts), occupational health services, and private services. It is decentralized and has multiple funding resources (e.g., the state, municipalities, households, voluntary private, and state national health insurance organizations). Hospital districts guide and control the development of imaging services, and other special services provided by municipal healthcare research, development, and training activities of coordinated municipal health information systems (9, 12, 13).

Finland utilizes an electronic Reporting System for Safety Incidents (RSSI) in Health Care Organizations (HaiPro, Awanic Ltd 2015c). The system was developed in 2006 and launched in 2007. The aim of the RSSI is to increase the documentation of incidents and to utilize this data to improve the quality and safety of care (14). Staff report medical and nursing treatment incidents, including near-misses. A further aim of the system is to report, analyze and learn of incidents, near-misses and patient safety risks, and to improve patient care processes. The RSSI is designed for internal use in healthcare units (university and local hospitals, social units, and health centers) and it is accessed online *via* the organization's intranet. Use of the system is voluntary, anonymity, and confidentiality are guaranteed, and no one is blamed. It provides information on the data reported, how local organizations have learnt from their incidents, and how the process of patient safety has been improved. The Finnish Society for Patient Safety (FSPS) manages the research permit practices related to the patient incident reports (9, 15, 16).

Finnish healthcare professionals can report different kinds of safety incidents, including medical imaging errors. The data are collected using web-based forms that contain predefined selection lists and spaces for free text (14, 16, 17). At present, RSSI data are mainly collected on the local and organizational level and are not analyzed or utilized on the regional or national level (9, 14–16).

The purpose of this study was to characterize the medical imaging reports of incidents on the national level. To our knowledge, this has not previously been studied in Finland. Our aim was to obtain information on near misses and adverse events of medical imaging. In addition, we wanted information on how the recurrence of incidents were prevented.

## Materials and Methods

### Data Collection

This study was an applied research that aims to highlight the practical problems in patient safety and find solutions to them. Awanic Ltd collected the data from a web-based national error-reporting database and sent it to the researcher (first writer). The data comprised safety incidents (*N* = 7,409) related to medical imaging that posed risks to patient safety in 2007–2017. The study involved 125 radiology departments from 18 regional and university hospital or other health service districts (Awanic Ltd).

The FSPS granted permission to use the medical imaging error data in the present study. In Finland, a registry study does not require ethical permission. The study followed the basic principles of research ethics (e.g., anonymity, confidentiality). The RSSI database is not linked to other databases, such as patient records. Disclosure of the data is subject to permission. As participation was voluntary, the medical units had the option of not participating in the study.

### Data on Medical Imaging Incidents

Incident reports (*N* = 7,409) were collected from January 2007 to December 2017, and 7,287 reports were included. Reports on nuclear medicine, radiotherapy, internal tests, duplicates, and reports not associated with imaging (*n* = 122) were excluded. All medical imaging-related notifications were included, even if the informer was not imaging staff (e.g., nurse, physician, secretary, or other). As some of the informers had not filled in every section of the reports, some information was missing. However, forms were included if the most relevant sections had been completed.

**Figure 1:**
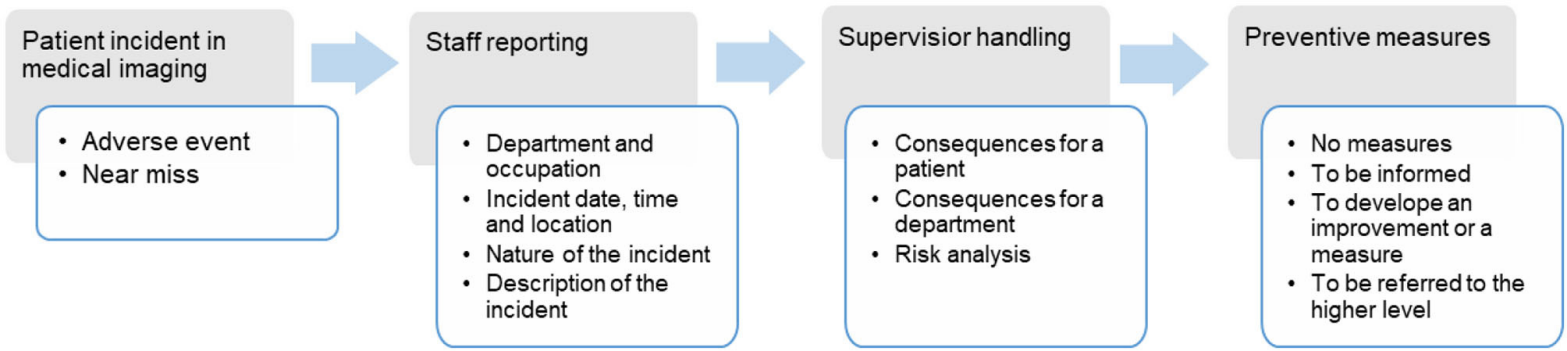
The sequence of the patient incident reporting process.

The information on the web-based forms and in the free-text descriptions that was related to medical imaging incidents were analyzed. Figure 1 presents the patient incident reporting process. The following information was reported: date, time, place (e.g., medical imaging) of the incident, and the unit of the person reporting. It was also possible to report the nature of the incident (adverse event, near miss) and the incident type, and to more specifically describe the event and how it occurred, the consequences for the patient and the unit, the conditions at the time of the incident, and other contributing factors. The staff also classified the causes of the incidents into eight main categories; drug or contrast agent, information flow or information management, other treatment or follow-up, imaging (not further specified), device or its use, accident/injury/damage, an invasive procedure, or diagnosis related.

The supervisor, usually the radiographer in charge or senior radiologist reviews the incident report and assigns a level of risk according to pre-defined categories: insignificant, low, moderate, high, and extreme. The supervisor also includes the contributing factors of the incident and describes measures to be taken to prevent the event reoccurring: (1) no measures, (2) informing of the event, (3) submitting it to a higher authority for a decision, or (4) designing improvement measures (14, 15).

The data were processed statistically using Microsoft 365 Excel (2016) and IBM SPSS statistics 25 (SPSS Inc., Chicago, IL, USA) for Windows. The results were reported using frequencies, percentages and cross-tabulation. The qualitative data will be analyzed in more detail in a subsequent article.

## Results

### Identification of Adverse Procedural Incidents Associated With Medical Imaging

This study included 7,287 adverse incidents that affected the safety of patients undergoing radiological procedures. The number of adverse incidents reported by staff rose (from 2007) by 10 per annum to 1,438 per annum over 10 years, making an average annual increase of 21.2%. Of the incidents reported, 75% concerned injuries to patients and 25% were near misses. Most of the patient injuries (28.9%) and near-misses (35%) were associated with the wrong patient, study or procedure. The other reasons for near miss reporting were for instance: data flow and data management (30%), malfunction of device (10%), error of radiology report or diagnosis (6%), and referral error (3%). The other reasons concerning patient injuries were: related to medication or contrast agent (17%), data flow and data management (15%), malfunction of device (14%), error of radiology report or diagnosis (10%), and referral errors (2%).

The most common consequences of the near-misses and adverse events were minor harm (37.2%) or no harm (27.9%). The most serious effects on patients were moderate harm, at 7.7% (563 cases), and serious harm, at 0.8% (61 cases). In 10.5% of the events, the consequences were unknown (e.g., a patient moved to another ward) and 15.8% were not assessed at all (missing). During 2007–2011, the consequences of events were assessed only occasionally, but from 2012, more regularly. The most common injuries to the patients were unnecessary or excessive radiation (wrong patient, side, or site), allergic reaction, burn, infection, falling, fainting, pain, aches, extra dose of contrast, and contrast media to tissue.

After the staff has assessed the consequences, supervisors assess the risk posed by the incident. The risk analysis matrix allows a supervisor to select the risk category. A supervisor selects the likelihood of an event: rare, unlikely, possible, likely, and almost certain; and its typical consequences: very minor, minor, moderate, major, or severe. These are divided into different risk types: insignificant (e.g., data flow or data management) low (e.g., equipment malfunction), moderate (use of contrast agent or accident), high (unexpected reaction of the patient), and extreme risk (serious injury to the patient). A supervisor may assess the incident as rare and its consequences very minor, in which case its risk type is insignificant or the probability of incident almost certain with severe consequences in which case the risk is extreme.

**Figure 2:**
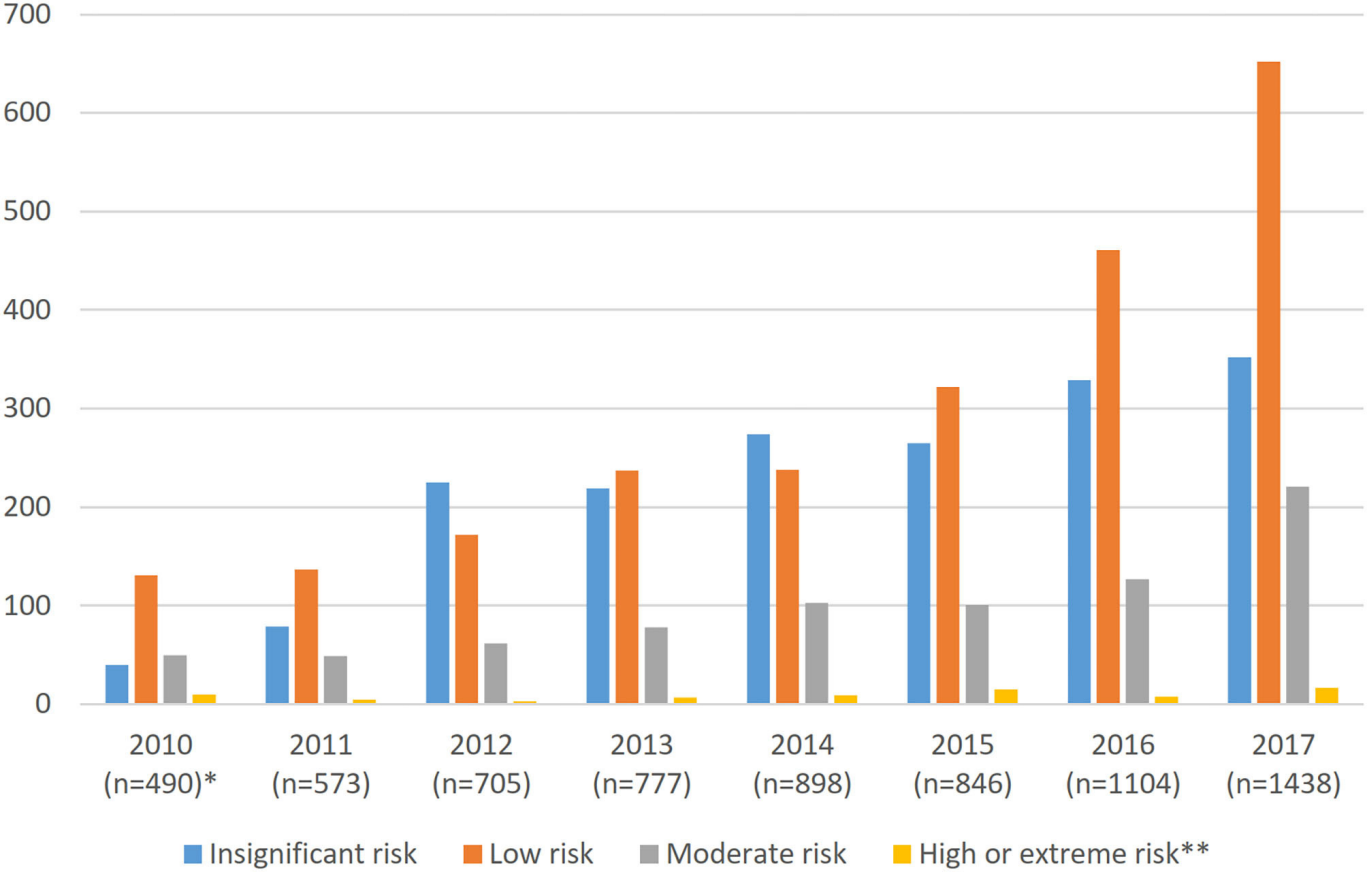
Annual reports and risk assessments in medical imaging in Finland 2010–2017. *In 2007–2008, the risks were not assessed, and in 2009 only 14 were assessed, so these years are not included in the figure. **High and extreme risks are combined (*n* = 17).

Supervisors assessed 69.4% (5,011 in total) of the patient risks, although 30.6% (about 200 per year) were not evaluated. In 2010–2017, 1.3% (66) of all risks were assessed as high and 0.2% (10) as extreme. Risks were generally estimated as low (47.7%), insignificant (35%), or moderate (15.7%). Figure 2 presents the annual distribution of the reported incidents by risk level and the number of reports during 2010–2017. In 2007–2009, the risk assessment was limited (14 reports) and are thus not taken into account in this figure.

**Figure 3:**
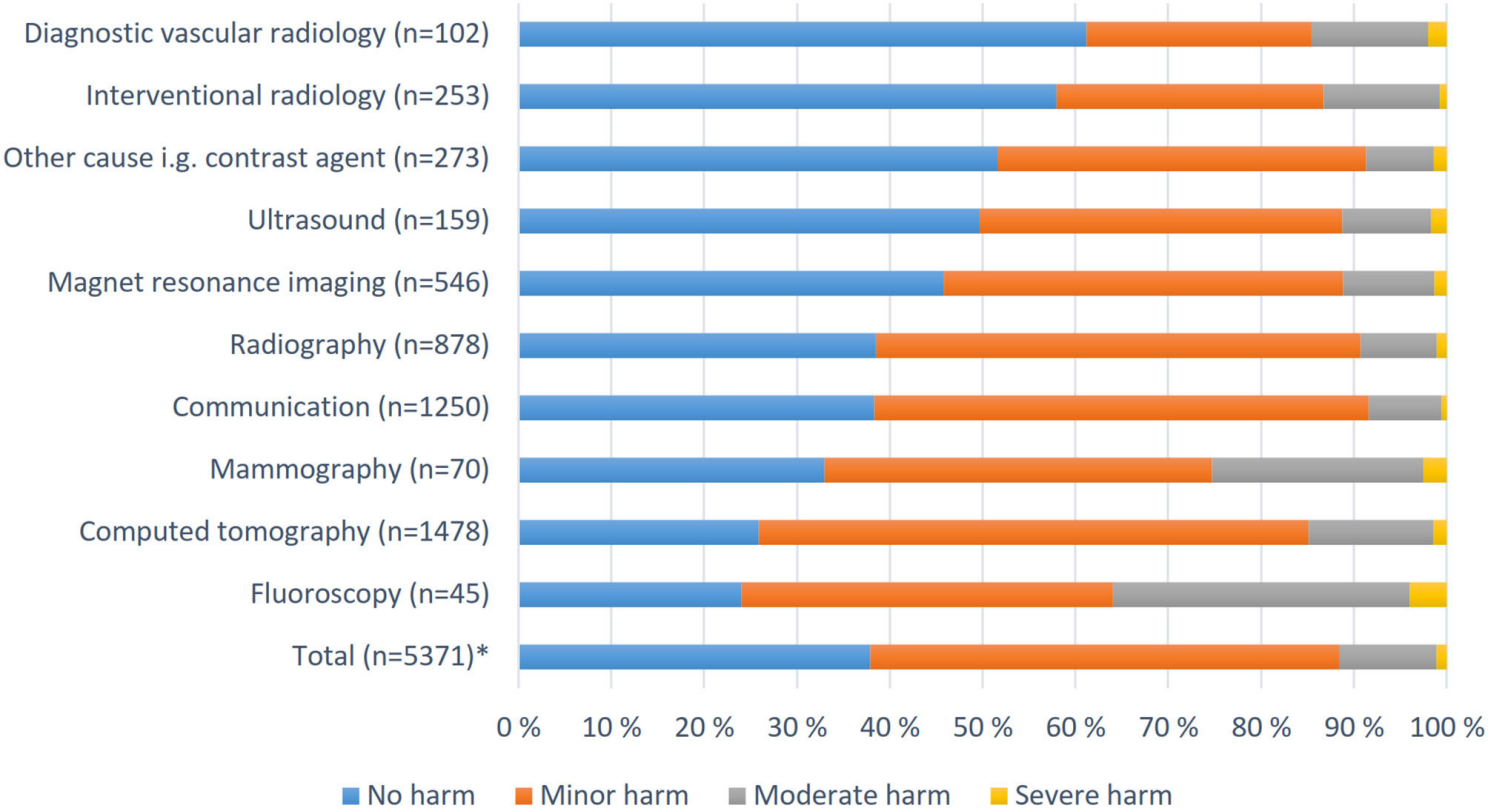
Patient injuries assessed by supervisors in different modalities and other contexts in Finland 2010–2017. *Missing or unknown 26.3% (*n* = 1,916).

The highest number of patient harm-related reports concerned computer tomography CT (27%); the second, problems with computers and telecommunications (25.6%); and the third, plain radiography (16.9%). Figure 3 shows how the consequences for patients were distributed among different modalities, communications, and other causes. Most of the severe and moderate harm cases occurred in fluoroscopy (2.8%) and mammography (2.1%). Approximately 73% of CT scan-related injuries were no or minor harm, and 15% were moderate or severe.

### Incident Reporting by Staff

Most incidents were reported by radiographers (62.1%), other nursing staff (e.g., practical nurse) (21%), other staff (e.g., office worker) (7.9%), and radiologists or other physicians (7.4%). Event types were evaluated in 7,083 reports (97.2%). Staff assessed near-misses and adverse events using the RSSI form's predetermined categories. About 26% of the patient consequences were not assessed at all or were reported as unknown. This is probably due to the fact that the informer is not working on radiology unit or the patient moves to another ward so the late effects of the event remain unknown. The most frequently reported type was associated with imaging (40%), but this was not further specified. A total of 20.1% events were related to information flow or information management, 13.9% concerned devices, and 13.4% were related to contrast media. Staff reported fewer events related to patient care or follow-up (4.6%), accidents or injuries (4%), diagnostic events (2.3%), and invasive procedures (1.8%).

Staff did not assess the extent of the risk. Supervisors estimated risks on the basis of the narrative content of the report and the risk matrix. Assessments by nursing supervisors showed that the patient was most likely to be at a low (40.7–50.3%) or insignificant risk (27.6–36.3%). The share of moderate risk was from 14.3 to 26.3%, high risk under 2% (0.9–1.9%), and extreme risk under 1% (0–0.1%). Medical supervisors estimated that the patient was at a high risk in 3.5% and at an extreme risk in 1.6% of the cases.

### Supervisors' Proposals for Measures

**Figure 4:**
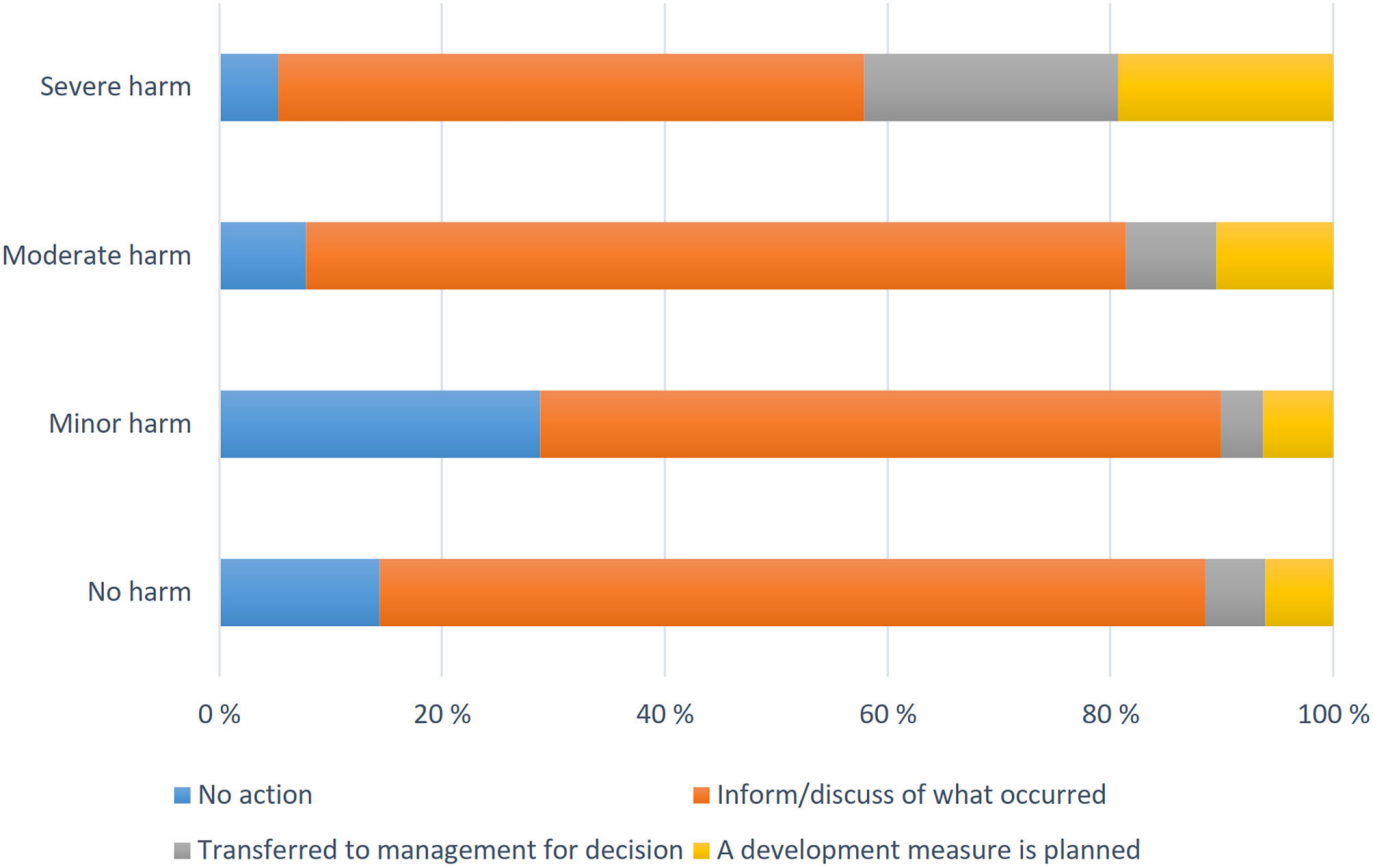
Reported patient harms with proposed measures in 2007–2017 in Finland.

A measure to prevent incidents was proposed by 89.4% of the supervisors. Generally (58.1%) supervisors suggested discussing in staff meetings. In under 6% (5.7%) of incidents improvement in working methods were recommended and in 5.4% of cases reports were delegated to a higher authority. Figure 4 shows the distribution of these measures in relation to the patient's incident assessment. In all categories, the most common recommendation by the supervisors was to inform the parties that were involved (patient, ward, attending physician) and to discuss about the incident with staff (52.6%). In the severe harm category, 22.8% were submitted to a higher authority and 19.3% to plan an improvement measure. The most general solution with minor harm category was “no action” (30%) and for preventing a recurrence of the event recommended, “informing of and discussing” (67%). When the patient injury was assessed as minor (e.g., extravasation during intravenous injection), it was classified as “no action” category.

## Discussion

In this study, we analyzed what kind of medical imaging errors were reported in Finland, how these incidents and consequences affected risks, and what measures supervisors took to improve patient safety. The strength of the study is that the data were large and comprehensive, covering 11 years. The average annual number of radiology examinations in Finland is 2.5 million, which is 1,084 examinations per thousand inhabitants. The percentage of reported patient adverse events is low, only 0.06% of radiology examinations per year. It is likely that only a part of the incidents were reported also in this study, as previous studies have found that some cases always remain unreported (2, 18).

In about a quarter of the cases, the injuries to the patient had not been assessed. This result support those of international studies that have identified difficulties in reporting because the impact on the patient is unknown or the informer is from another unit, in which case the information obtained from the event is inaccurate (19–23). In the initial years, 2007–2011, forms were only filled in occasionally, but this clearly increased in later years. The probable reason for this is the greater national attention paid to patient safety in Finland due to the Patient Safety Implementation Plan (341/2011). This has increased the identification and reporting of incidents, and more hospitals joining the RSSI (24). Our research shows that when staff were encouraged to fill in adverse incident forms, the number of patient safety incident reports increased by 20% annually. Therefore, we cannot directly conclude that the number of complications increased every year.

In 2015, 624 radiologists and 3,262 radiographers were working in Finland. In this study, most of the reporters were radiographers and other nursing staff. Other physicians and radiologists reported only 7.5%. In other Finnish studies the number of incident reports made by physicians have been 2.5–10% (9, 17). Our result is comparable to those of previous international studies that have found that physicians make fewer incident reports (25, 26) and most patient incident reports are made by nursing stuff (19, 30). The physicians feel more obliged to deal with serious cases than reporting near misses (19, 20, 30) whereas nurses are more diligent and follow the instructions (26).

Supervisors are provided with guidelines on how to handle notifications. These guidelines offer advice on selecting from the different options the correct type of event, the consequences for the patient, the unit, the correct risk category, and suggested development measures. The event should be evaluated according to general practice, not on the basis of the individual patient. This means that the risk assessment must take into account the wider context of the individual case, which can be challenging. Therefore, supervisors may assess risks as insignificant or minor, even if the patient was harmed. Radiologists reported more serious harm (2.2%) than radiographers in charge (0.7%) did, and their estimated risk levels were higher than those of the other staff. This result is consistent with that of previous studies (23, 25, 26) that have found that physicians usually report the most serious patient injuries (severe injury or infection, death). In Finland this is probably due to the fact that the most severe cases are referred to a radiology supervisor.

During the initial years, both physicians' and radiographers' information technology (IT)-related notifications were relatively high in number. The number decreased each year as IT problems were solved. There are usually problems when starting a new operation, and knowing this risk, having a contingency plan to investigate operational faults, and striving to remedy them as soon as possible are essential.

On average, 10.5% (5.6–13.6%) of patient injuries related to modalities were unclassified because the person filing the report did not know what had occurred to the patient after the examination. Our previous study of patient injury reports due to imaging (27) provides more information on these incidents. Usually for example, in interventions, patient injury is noticed immediately, but in other modalities, the effect of the event may become apparent later.

In CT, 24.3% of incidents were related to contrast agents, due to leakage of the contrast media into the surrounding soft tissues instead of the normal intravascular compartment, failed injection (the cannula came off or contrast agent was injected into tissues or the contrast agent syringe did not work), or patients' allergic reactions. Contrast media extravasation (CMEV) is a well-known complication in CT scanning. Most extravasations cause minimal swelling or erythema, but skin necrosis may also occur (28). Therefore, efforts should be made to reduce the migration of the contrast medium into the tissue: particularly in CT scanning, where the injection rate and volume of contrast medium is high.

The modalities most involved in patient incidents were CT, radiography, and MRI. Our results are consistent with those of other studies (2, 27, 29), with the exception that in this study, MRI incidents were more common than ultrasound incidents. This is probably because in Finland, more contrast-enhanced MRI scans are performed than contrast-enhanced ultrasounds. The higher rates of moderate harms in fluoroscopy and mammography were related to information flow, incomplete or unclear information and equipment problems. Severe harms, such as serious infection or accident, were related to invasive procedures.

This study has some limitations. First, the classification of adverse events and near-misses was based on the incident description, which was occasionally limited and not all sections of the form had always been completed. Therefore, the “not known” and “missing” category made up 20–30% of incidents. Second, as only a small proportion of incidents are reported, and even less near-misses (4–7), our data most likely represents only a part of the actual problems in incident reporting. Third, because the RSSI form does not contain own category for imaging staff, the exact number of incidents cannot be measured. Fourth, the results can only be generalized to a certain extent, as they were collected in one country. And fifth unfortunately, the supervisors' development proposals for resolving the incident that had occurred were in written form, and due to the large amount of data, could not be discussed in more detail in this article.

In our study, the number of reported near-misses was lower (26.3%) than the number of incidents (72.6%). This may be because incidents are easier to identify and staff consider it more important to report events that have consequences for the patient. It is important to note that when we invest in patient safety by collecting data on near-misses, we can also use them in effect on damage prevention (30, 31).

Supervisors planned some sort of measure to prevent the recurrence of incidents in almost 90% of cases. The most common solution, at over 50%, was discussion with the staff. Conversation is a quick and easily organized solution, but in our opinion not necessarily the most effective. It is good practice and can solve the problem, but if the incident is repeated, it would be better to investigate adverse event's root causes. It is important to notice that even when the patient was not harmed some supervisors planned a development of operational safety. It is good that all cases are taken seriously.

The more severe the damage, the higher the proportion of improvement measures and notifications at a higher level. It is common that when the harm is assessed as severe (serious injury or death of the patient, resource shortage or IT problems) the proportion of improvement measures and notifications are submitted to a higher authority (30). This is probably due to the desire to receive support and at the same time inform the senior management of the most serious events. Senior management should also answer questions about resourcing and communicate with equipment and other suppliers.

## Conclusion

Improving patient safety requires timely, accurate and clear reporting of various patient safety deviations. Therefore, it is important that national data on patient safety incidents are collected and analyzed. Based on the incident reports, supervisors should provide feedback to staff, develop a plan to prevent accidents, and monitor the impact of the measures taken. Information on the development of operational safety should be disseminated widely to all healthcare professionals so that others can avoid making the same mistakes and improve their own patient safety by learning from others.

## Implications for Practice

- To prevent incidents, near misses should be reported more frequently.
- Information on measures taken to improve operations needs to be more widely disseminated.

## Data Availability Statement

The datasets presented in this article are not readily available because, the data that support the findings of this study are available from the Finnish Society for Patient Safety but restrictions apply to the availability of these data, which were used under license for the current study, and so are not publicly available. Data are however available from the authors upon reasonable request and with permission of the Finnish Society for Patient Safety. Requests to access the datasets should be directed to https://tarja.tarkiainen@ppshp.fi.

## Ethics Statement

Ethical review and approval was not required for the study on human participants in accordance with the local legislation and institutional requirements. Written informed consent for participation was not required for this study in accordance with the national legislation and the institutional requirements.

## Author Contributions

TT analyzed and interpreted the data together with JN. MT and SS verified and assessed the accuracy of the text. MH as a professional bio statistician verified the table and figures. All authors read and approved the final manuscript.

## Conflict of Interest

The authors declare that the research was conducted in the absence of any commercial or financial relationships that could be construed as a potential conflict of interest.

## Publisher's Note

All claims expressed in this article are solely those of the authors and do not necessarily represent those of their affiliated organizations, or those of the publisher, the editors and the reviewers. Any product that may be evaluated in this article, or claim that may be made by its manufacturer, is not guaranteed or endorsed by the publisher.

## Abbreviations

FSPS: The Finnish Society for Patient Safety
MERS: Medical Error Reporting System
RaER: the Radiology Events Register
RSSI: Reporting Systems for Safety Incidents.
